# Supplementary material for: Mechanical properties of a polylactic 3D-printed interim crown after thermocycling
Source: PLoS One. 2025 Jan 30;20(1):e0318217. doi: 10.1371/journal.pone.0318217 (PMC11781676; doi:10.1371/journal.pone.0318217)
Supplement: Attachment — Submitted filename: FReponse letter for Plos one (2).docx [file pone.0318217.s005.docx]

<http://www.google.co.kr/url?sa=i&rct=j&q=&esrc=s&source=images&cd=&cad=rja&docid=VI0FkyAy_B2MMM&tbnid=DIJN1x1lf4AisM:&ved=0CAUQjRw&url=http://blog.daum.net/loveubrad/3&ei=MxmpUq2kJs2fiAf4t4CoAg&bvm=bv.57799294,d.aGc&psig=AFQjCNGPIXzBVP6rLjHPpnbHaf0LBMjETg&ust=1386900141258164>Dept. of Advanced General Dentistry

Yonsei University College of Dentistry

250 Seongsanno, Seodaemun-gu

Seoul 120-752, Republic of Korea

1. Title:

2^nd^ REVISION: [PONE-D-24-44087R1] - [EMID:b37b4b5ffb7c3b2c]

**Title:** **Mechanical properties of a polylactic 3D-printed interim crown after thermocycling**.

2. Corresponding author’s name, mailing address, phone number and fax number:

Prof. Bock-Young Jung

E-mail address: jby1004@yuhs.ac

Phone: (+82)2-2228-8980, Fax: (+82)2-2227-8906

3. Names of authors:

Re-Mee Doh, Won-Il choi, Seo young Kim, Bock-Young Jung

This 2^nd^ response letter accompanies our online submission of the manuscript titled “Mechanical properties of a polylactic 3D-printed interim crown after thermocycling”.

We sincerely appreciate the reviewer’s valuable comments, which made our manuscript sounder and more reasonable. We are resubmitting the manuscript after performing the revisions requested by the reviewers. We have tried to address all of the comments and issues raised by the reviewers carefully and have highlighted all of the changes in yellow in the revised manuscript. We sincerely hope that our revised manuscript meets the high standards of your journal**.**

We hope that this paper is now suitable for publication in PLOS ONE.

We thank you in advance for your time and attention.

Sincerely yours,

8^th^ January, 2025

Bock Young, Jung

Response letter

Manuscript ID: [PONE-D-24-44087R1] - [EMID:b37b4b5ffb7c3b2c]

MS-Type: Original research

Title: "Mechanical properties of a polylactic 3D-printed interim crown after thermocycling."

Corresponding Author: Bock-Young, Jung

**Comments to the Author**

**Reviewer #1: I appreciate the answers to my questions. The effort was worth it and the manuscript is much better and more complete.**

**Reviewer #2: The authors have adequately addressed my comments and suggestions. The manuscript has improved. In my opinion, it is suitable for publication.**

**Additional Editor Comments:**

**1. Please present reference(s) for every sentence in introduction section and also in discussion section.**

**Response:** Thank you for the comments.

We have carefully reviewed the Introduction and Discussion sections to ensure that all external research findings and information, apart from the results of this study, have been appropriately cited with references.

**2. Please add reference(s) for this sentence: The parameters applied to this investigation were the values that have been recommended in previous studies or by the manufacturers, including the layer height, nozzle size, ejection speed, and curing time (Table 1)**

**Response:** Thank you for this comments.

We have added the references at their appropriate positions in the Introduction and Discussion sections as requested. No additional references were added. The reference numbers have been adjusted according to the citation order in the main text.

**Text changed:**

The parameters applied to this investigation were the values that have been recommended in previous studies [17-19] or by the manufacturers, including the layer height, nozzle size, ejection speed, and curing time (Table 1).

**3. The paragraph in line 249-261 needs references. You mentioned ‘’previous studies’’ however any studies were cited. Please include citations for Alharbi, Osman, and the study recommending 30° printing orientation.**

**Response:** Thank you for the comments.

To enhance clarity, we have now cited each reference individually at the specific points where they are mentioned. Consequently, the reference numbers have been adjusted to reflect the citation order in the main text.

**Text changed:**

This study aimed to compare the mechanical properties of PLA with those of conventionally used CAD/CAM interim dental restorations. The parameters in this investigation were based on recommendations from previous studies and manufacturers[17-19]. For PLA, postprocessing annealing is the main process for improving mechanical properties; hence, the bed temperature is usually kept above the glass transition temperature (Tg, approximately 60°C) to maximize bonding between the deposited layers[25]. In this study, the nozzle temperature and bed temperature were maintained at approximately 200°C and 65°C, respectively, to maximize the balance between the degree of crystallinity and postprocessing annealing. Previous studies have explored the effects of the printing angle on the mechanical properties of 3D-printed interim dental restorations. Alharbi et al.[18] reported that a sample printed perpendicular to the load direction exhibited higher compressive strength, whereas Osman et al.[19] recommended 135° for DLP. Additionally, one study reported that printing at 30° resulted in the highest FS [17]. In this study, a 135-degree printing orientation for both PLA FDM and SLA was consistently used, based on prior research indicating that this angle was the ideal orientation for producing optimal mechanical properties.

Thank you very much again.

*We appreciate the reviewers’ comments, which have helped us to improve our manuscript.*

Sincerely,

Bock Young Jung
